# Outcome reporting in studies of paediatric achalasia: A systematic review

**DOI:** 10.1002/jpn3.70128

**Published:** 2025-06-22

**Authors:** Jonathan J. Neville, Sierra Schaffer, Simon Eaton, Nigel J. Hall

**Affiliations:** ^1^ Great Ormond Street Institute of Child Health University College London London UK; ^2^ Department of Paediatric Surgery Royal Alexandra Children's Hospital Brighton UK; ^3^ University Surgery Unit University of Southampton, Faculty of Medicine Southampton UK

**Keywords:** achalasia, core outcome set, gastroenterology, outcome measures, surgery

## Abstract

**Objectives:**

Paediatric achalasia is a rare condition associated with significant morbidity. A core outcome set (COS) would standardise reporting, enable comparison of data sets, and focus research efforts; ultimately improving care for children with achalasia. We aimed to identify outcomes currently reported in studies of paediatric achalasia to inform outcomes for a COS.

**Methods:**

A systematic review was performed in accordance with the Preferred Reporting Items for Systematic Reviews and Meta‐analysis guidelines. Studies investigating children ≤18 years of age with a diagnosis of achalasia were included. Primary and secondary outcomes were recorded and assigned to OMERACT core areas. The study was pre‐registered (PROSPERO: CRD42024509855).

**Results:**

Sixty‐two studies were included in this review, consisting of 54 retrospective and 8 prospective studies. Median cohort size was 20 patients (inter‐quartile range: 13–28). Forty‐eight unique outcomes were reported. The most common outcomes reported were intra‐operative complications (65%, 40 studies), post‐operative complications (58%, 36 studies) and length of stay (58%, 36 studies). A primary outcome was specified in 12 studies (19%), the most common was the Eckardt score (13%) in 8 studies. Studies least frequently reported outcomes in the death (21%, 13 studies) and pathophysiological manifestations (35%, 22 studies) core areas.

**Conclusions:**

The studies included in this review were predominantly small and retrospective. Of the few studies that specified a primary outcome, the majority used the Eckardt score, which is unvalidated in children. Outcomes relevant to pathophysiological manifestations, life impact and survival were under‐reported. A COS for paediatric achalasia, involving key stakeholders, would ensure that patient‐relevant outcomes were reported, reduce heterogeneity and facilitate meta‐analysis.

## Abstract (highlights)

**What is Known:**

- Achalasia is a rare disease in children associated with significant morbidity.
- Core outcome sets (COSs) are valuable in rare paediatric conditions because they standardise reporting, enable meta‐analysis and focus research efforts.

**What is New:**

- Studies often use the Eckardt score as a primary outcome, but this is unvalidated in children.
- Outcomes relevant to the pathophysiological manifestations of achalasia and its impact on a child's quality of life are under‐reported.
- This study will inform potential outcomes for the development of a COS in paediatric achalasia.

## 1 INTRODUCTION

Achalasia is a rare disease of oesophageal dysmotility that affects 0.11–1.80 per 100,000 children.^1^, ^2^ Disordered oesophageal peristalsis, high resting tone of the lower oesophageal sphincter (LOS), and failure of the LOS to relax on swallowing cause affected children to develop progressive dysphagia, vomiting, retrosternal pain and impaired growth.^1^ Treatment options are limited and aim to reduce symptoms by lowering LOS pressure. These include medical therapies, endoscopic treatments and surgery.^3^ Evidence for an optimal management strategy of children with achalasia is lacking. To date, no large prospective comparative trials have been undertaken in children.^2^, ^3^, ^4^, ^5^ Similarly, little is known about how achalasia, and its treatment modalities, impact on a child's quality of life (QoL).^6^ There can be no doubt that a greater understanding of the relationships between oesophageal physiology, treatments and outcomes is necessary to improve the care provided to these children. However, before conducting studies comparing treatments in children with achalasia, it is necessary to identify the most important outcome measures for inclusion.

Core outcome sets (COSs) are standardised outcomes that can guide further research and facilitate data pooling and meta‐analysis.^7^ The development of COS in rare paediatric disease is essential to allow future studies to report important and comparable outcomes that are relevant to children.^8^ No COS has been developed for patients with achalasia, and current measures of treatment success in achalasia, such as the Eckardt score, are not applicable to children.

To inform the creation of a COS for children with achalasia, we performed a systematic review to determine which outcomes are currently reported in clinical research studies relating to children with this condition.

## 2 METHODS

A systematic review of the literature was performed according to the Preferred Reporting Items for Systematic Reviews and Meta‐analysis guidelines.^9^ The study protocol was specified in advance and registered on PROSPERO (CRD42024509855).

An electronic database search was performed of MEDLINE, Web of Science and the Cochrane Library from January 1990 to December 2023 (Table S1). Reference lists were also searched. Prospective and retrospective studies, including published protocols, investigating children ≤18 years of age with a diagnosis of achalasia were eligible for inclusion. No criteria for a diagnosis of achalasia were specified. Review articles, case reports, case series including ≤5 patients, clinical guidelines and articles not published in English were excluded. Studies that included mixed populations of children and adult patients (>18 years of age) were also excluded.

Two reviewers (JJN and SS) independently screened the titles and abstracts of each study identified from the literature search. Articles that did not meet the inclusion criteria and duplicates were excluded. The full text of the remaining articles was assessed against the inclusion criteria. Disagreements were resolved by a third reviewer (NJH). Data were extracted by JJN and validated by SS independently. Publication date, study design, patient population, sample size and reported outcomes were extracted.

An outcome was included if it was discussed in the methods or results of the study. Primary outcomes were identified when referred to as the ‘primary outcome’ in the study text. Definition of the outcome and the method for measuring each outcome were also extracted. Inter‐study heterogeneity in the definition and measurement of outcomes was assessed via descriptive analysis. The outcomes reported in prospective studies were highlighted as these studies were expected to have more rigorous methodology. Outcomes with similar meanings were summarised as outcome terms to account for heterogeneity in reporting.

Each outcome term was assigned to an OMERACT 2.0 core area.^10^ The OMERACT 2.0 filter aims to ensure a breadth of outcomes are recorded when developing a COS. The core areas are death, life impact, resource use and pathophysiological manifestations. Adverse events should be reported across all four core areas; however, we included adverse events as a fifth core area for this systematic review. Outcomes were allocated to a core area by two reviewers independently (JJN and SS), with disagreements resolved by a third reviewer (NJH). The distribution of outcome terms across each core area in each study was assessed.

Primary outcomes were assessed using the COSMIN risk of bias tool according to the COSMIN methodology for systematic reviews of patient‐reported outcome measures (PROMs).^11^ For each domain assessed, the primary outcome reported in each study was scored on a 4‐point Likert scale; 1—*very good*, 2—*adequate*, 3—*doubtful* and 4—*inadequate*. Mean scores were derived per study. Data analysis was descriptive; no quantitative data were collected from the included studies, so no meta‐analysis was performed.

## 3 RESULTS

**Figure 1:**
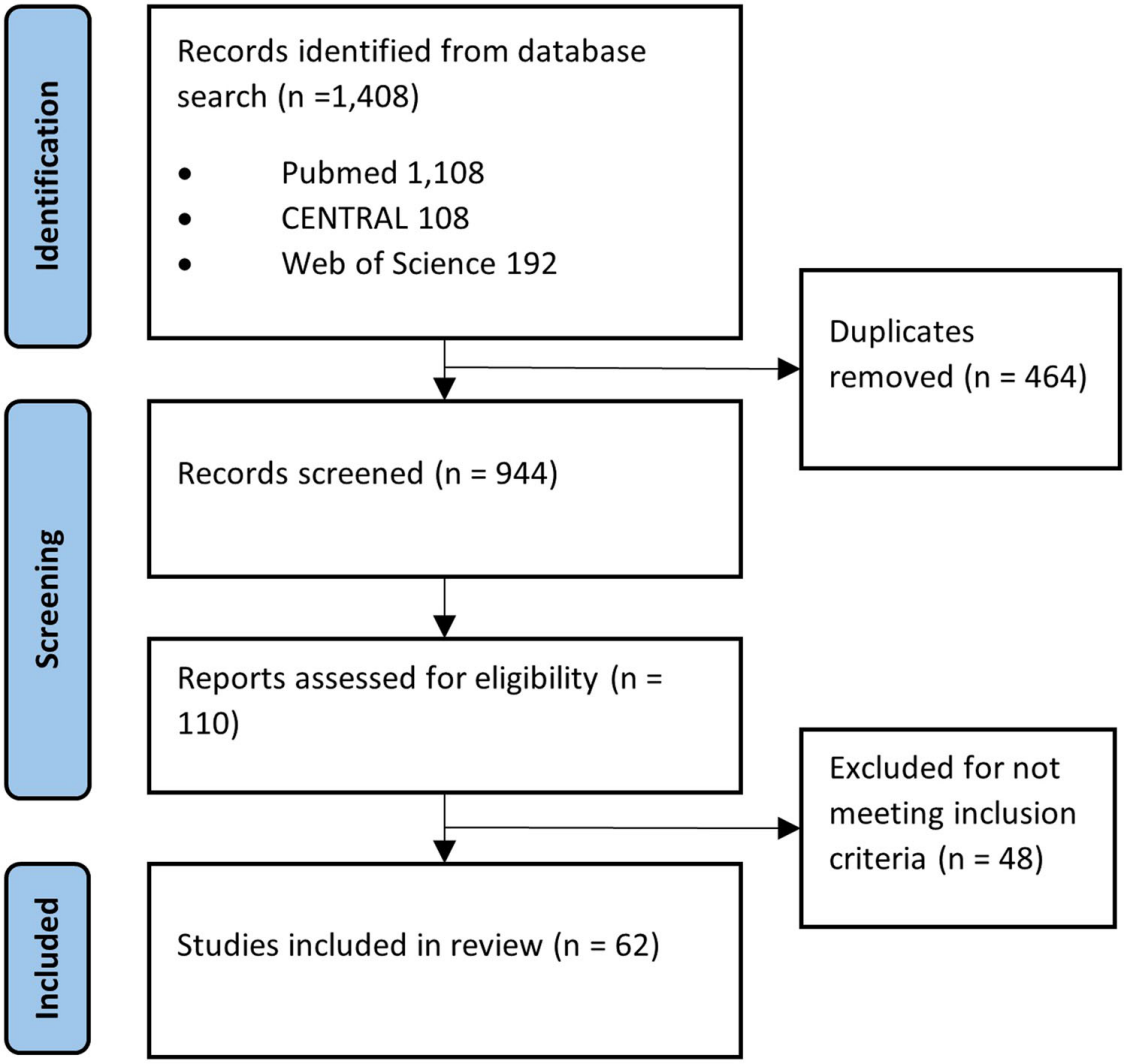
PRISMA flow diagram. PRISMA, Preferred Reporting Items for Systematic Reviews and Meta‐analysis.

In total, 1408 articles were identified from electronic database searching (Figure 1). After duplicate removal, 1190 studies underwent title and abstract screening. The full texts of 110 studies were reviewed, and 62 met the inclusion criteria. No additional studies were identified from the search of the reference list. All 62 studies were included in this review.

Of the 62 studies, 54 (87%) were retrospective and 8 (13%) were prospective (Table S2). There were no randomised control trials. Median cohort size was 20 children (range: 9–130). Age inclusion criteria varied, but studies most commonly included patients aged 0–18 years (16%, 10 studies). Studies were from Europe (42%), North America (24%), Asia (21%), South America (5%), Africa (5%) and Australasia (2%). One study, which included 117 children, was an international collaboration of 14 centres across North America, Europe and Asia.^12^

A total of 48 unique outcomes were reported. The most frequently reported outcomes were intra‐operative complications (65%, 40 studies), post‐operative complications (58%, 36 studies) and post‐operative length of stay (58%, 36 studies). A primary outcome was specified in only 12 studies (Table S2).^6^, ^12^, ^13^, ^14^, ^15^, ^16^, ^17^, ^18^, ^19^, ^20^, ^21^, ^22^ The most frequently reported primary outcome was the Eckardt score (13%, eight studies). Two studies reported treatment failure as the primary outcome, defined as the requirement for subsequent treatment, with one investigating the survival time to treatment failure. One study reported QoL as the primary outcome, assessed by the Pediatric Quality of Life Inventory (PedsQL) tool. A further study used post‐operative manometry features as the primary outcome.

Eight prospective studies were included.^6^, ^13^, ^14^, ^15^, ^23^, ^24^, ^25^, ^26^ Of these, only four reported a primary outcome: three used the Eckardt score, and one assessed QoL using the PedsQL. The most commonly described secondary outcomes were length of stay, intra‐operative complications, procedure duration, symptom resolution and post‐operative manometry features, all reported by four studies each.

Heterogeneity was identified in many outcome definitions and timepoints at which outcomes were assessed (Table S3). Most notably, different definitions were used for symptom recurrence following an intervention. The majority of studies referred to the presence of any symptoms post‐intervention, reported by the patient, as symptom recurrence. Meyer et al. defined symptom recurrence as daily symptoms, consistent with achalasia, interfering with QoL.^27^ Other studies stipulated that symptomatic improvement had to occur with the requirement for no further interventions.^19^, ^26^, ^28^ Mattioli et al. used a modified Visick symptom scale to classify post‐operative symptoms as absent, improved, unchanged or worse compared to pre‐operatively.^29^

Multiple different assessment tools were used to measure general and disease‐specific patient QoL. The most commonly used general QoL assessment tool was the PedsQL. Achalasia‐specific measurements included the paediatric assessment tool developed by Marlais et al. and the Achalasia Severity Questionnaire, which was developed in adults.^6^, ^30^ Definitions of post‐operative complications, such as the development of gastro‐oesophageal reflux disease and oesophagitis, differed between studies. Certain studies used patient‐reported symptoms or disease‐activity scores to diagnose gastro‐oesophageal reflux disease and oesophagitis; other studies used routine or non‐routine (symptom‐led) post‐operative investigations (Table S3).

**Figure 2:**
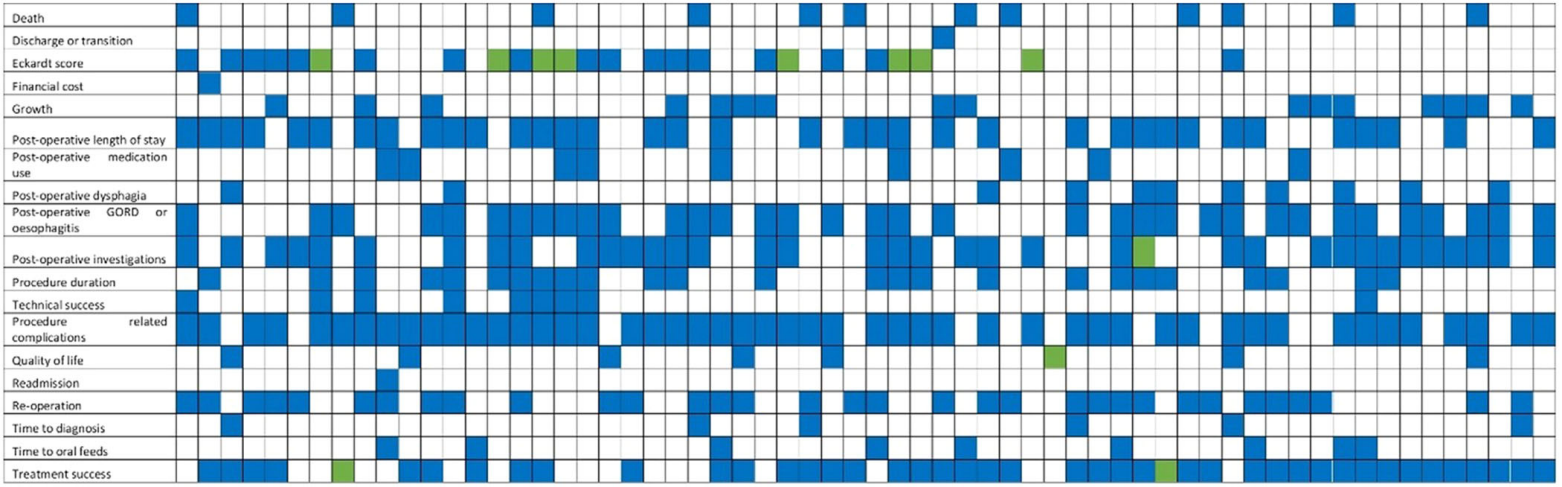
Outcome matrix of 19 outcome terms across 62 included studies. Each column represents a single study. Green signifies that the outcome was used as the study's primary outcome. Blue signifies that the outcome was used as a secondary outcome.

**Figure 3:**
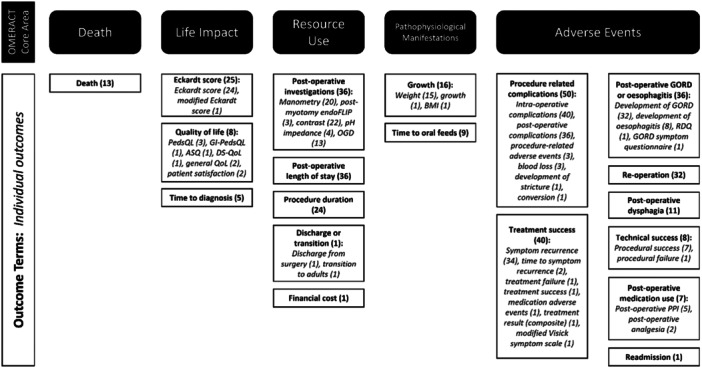
Reported outcomes (italics) mapped to outcome terms (bold) and assigned to OMERACT core areas. The frequency of studies reporting each outcome or term is indicated in parentheses. Note that one study might report multiple outcomes and terms.

To account for variation in definitions, outcomes were summarised as 19 terms (Figure 2). The most common outcome terms reported were procedure‐related complications (81%, 50 studies) and treatment success (65%, 40 studies). The 19 outcome terms were mapped to the five OMERACT core areas (Figure 3). Only one study allocated outcomes to all five core areas.^31^ The median number of OMERACT core areas to which outcomes were assigned was three. Two studies reported outcomes that mapped to a single core area (life impact and adverse events).^6^, ^32^ The core areas with the most mapped terms were adverse events (eight terms) and resource use (six terms). Two terms were mapped to pathophysiological manifestations and three terms to life impact.

The primary outcomes reported by 12 studies were assessed using the COSMIN tool (Table S4). All primary outcomes were scored as adequate. No studies performed a PROM development, pilot or cognitive interview study. No studies assessed the content or structural validity of any primary outcome. Similarly, no studies investigated the internal consistency or cross‐cultural validity of the primary outcomes used. All studies used a comparator instrument or reported secondary outcomes; however, the strength of these varied. No secondary outcomes directly cross‐validated the primary outcome.

## 4 DISCUSSION

We have conducted a systematic review of the literature to identify which outcomes are reported in studies investigating children with achalasia. We have observed variation in reported outcomes and the definitions and timepoints used. The majority of published paediatric achalasia research is retrospective single‐centre studies of small cohorts. There were no randomised controlled trials comparing treatment modalities. Few studies reported a primary outcome, and often the Eckardt score was used, which is not validated in children. Heterogeneity exists in the definitions of commonly reported outcomes, including assessments of QoL. Studies typically focused on technical procedural details, and outcomes most commonly mapped to the resource use and adverse events core areas. Few studies investigated outcomes mapped to the life impact and pathophysiological manifestations core areas. Overall study quality was rated as low.

The focus of many studies included in this review was short‐term procedural outcomes related to the treatment modalities in paediatric achalasia. This is evidenced by the most common reported outcomes (intra‐operative and post‐operative complications, and length of stay) and the majority of outcome terms mapped to resource use and adverse events. This highlights the technical focus of many studies published in paediatric achalasia. No studies have reported patient or public involvement and engagement in the design, management or analysis of the research. As such, a paucity of patient‐centred outcomes was reported, and few outcomes were mapped to life impact.

The Eckardt score was used in multiple studies as a primary or secondary outcome. However, its use as a measurement of treatment success is not validated in children. The Eckardt score grades the frequency of dysphagia, regurgitation and retrosternal pain symptoms, and the degree of absolute weight loss, on 3‐point severity scales (absent to occurs with every meal, and no weight loss to >10 kg).^33^ The use of absolute weight loss, as opposed to a change in weight *Z*‐score, makes the Eckardt score inapplicable and unvalidated, particularly in younger children. Similarly, studies have reported that the symptom profile of children with achalasia can be different to that of adults, with fewer reporting retrosternal pain.^2^, ^19^, ^34^ The Eckardt score requires modification and validation in children before it is used as an outcome measure. In addition, studies that assessed QoL also used disease‐specific scores validated in adults and not children.^35^, ^36^ Again, this may limit the applicability of these tools in paediatric achalasia. A disease activity score that is validated for paediatric achalasia that incorporates the impact of the disease on QoL should be developed.

A systematic review of the surgical management of achalasia was published in 2020 and included 33 studies.^2^ Three studies were prospective, and no randomised trials were identified. As in this study, the median cohort size was 20 children. Outcomes reported in this systematic review included symptom improvement, Eckardt score, weight gain, post‐operative manometry features and post‐operative contrast study findings. The authors noted significant heterogeneity in outcome definitions, which made evidence synthesis difficult. A lack of objective and comparable outcome measures for treatment success meant that the authors could only formally compare surgical results from 13 studies. This highlights the need for consistent outcome reporting.

The outcomes identified in this study will be used to inform the creation of a COS for paediatric achalasia.^37^ Each outcome will be considered for inclusion in a three‐stage Delphi consensus exercise involving healthcare professionals, patients, caregivers, and researchers. The importance of each outcome will be ranked in the Delphi exercise. Outcomes will then be discussed at a consensus meeting and considered for inclusion in the final COS. The COS will include the outcomes that are the most important to all affected by paediatric achalasia and will be used in future research.

To our knowledge, this is the first study to systematically analyse the outcomes reported in studies investigating children with achalasia. Strengths of this study include the robust methodology, and the large number of studies included. To ensure that this review included the maximum number of studies, we did not define criteria for the diagnosis of achalasia. Whilst it may be considered that this study is limited by the small cohort sizes of included studies, the paucity of prospective studies and the absence of randomised controlled trials, we do not believe these to have negatively impacted the aim of this review.

## 5 CONCLUSION

In conclusion, there is wide variation in the outcomes reported in studies of paediatric achalasia. Most outcomes reported are related to resource use and adverse events core domains. Few studies report patient‐focused outcomes within the life impact core domain. The evidence base consists predominantly of small, retrospective studies that focus on the technical success of interventions. Given the variation in outcomes reported, lack of consistent definitions and timepoints used, and concerns regarding the validity of certain outcomes in children, further work should focus on developing a COS for paediatric achalasia that is informed by this systematic review. Involving patients and families in the creation of a COS would ensure patient‐relevant outcomes were represented. Similarly, a paediatric achalasia disease activity score that is validated for use in children should be developed and used to inform treatment success and decisions regarding optimum treatment modalities.

## CONFLICT OF INTEREST STATEMENT

The authors declare no conflicts of interest.

## Supporting information

Table S1: Database search strategy.

Table S2: Characteristics of studies selected for inclusion.

Table S3: Variation in reported outcome definitions and measures between studies.

Table S4: Results of the COSMIN risk of bias assessment.
