# Supplementary material for: Outcome reporting in studies of paediatric achalasia: A systematic review
Source: J Pediatr Gastroenterol Nutr. 2025 Jun 22;81(3):523–9. doi: 10.1002/jpn3.70128 (PMC12408982; doi:10.1002/jpn3.70128)
Supplement: Supplementary file 1 — Table S1: Database search strategy. [file JPN3-81-523-s002.docx]

Supplementary Table 1: Database search strategy.

| **Database Search String** |
| --- |
| (child* or toddler* or adolescen* or teen* or juvenile* or p?ediatric*) AND  (achalasia [MESH] or ?esophageal achalasia) AND  (Heller* cardiomyotomy or Heller* myotomy or per oral endoscopic myotomy or POEM or endoscopic balloon dilatation or pneumatic dilatation) or (botulinum toxin AND achalasia) |
