# Supplementary material for: Outcome reporting in studies of paediatric achalasia: A systematic review
Source: J Pediatr Gastroenterol Nutr. 2025 Jun 22;81(3):523–9. doi: 10.1002/jpn3.70128 (PMC12408982; doi:10.1002/jpn3.70128)
Supplement: Supplementary file 2 — Table S2: Characteristics of studies selected for inclusion. [file JPN3-81-523-s001.docx]

| **Study** | **Year** | **Study design** | **Sample Size** | **Population** | **Primary Outcome** |
| --- | --- | --- | --- | --- | --- |
| Provenzano *et al*. Eur J Ped Surg | 2023 | Retrospective | 40 | 0 – 15 year olds | None |
| Delgado-Miguel *et al*. J Ped Surg | 2023 | Retrospective | 24 | 0 – 17 year olds | None |
| Saiad *et al*. J Ind Assoc Ped Surg | 2023 | Retrospective | 13 | 0 - 15 year olds | None |
| Guler *et al*. Turk J Gastroent | 2023 | Retrospective | 10 | 2 - 16 year olds | None |
| Srivastava *et al*. Ind J Gastroent | 2023 | Retrospective | 48 | 0 – 18 year olds | None |
| Rafeeqi *et al*. J Ped Surg | 2023 | Retrospective | 33 | 0 – 16 year olds | None |
| Samejima *et al*. Eur J Ped Surg | 2023 | Retrospective | 10 | 0 – 18 year olds | Eckardt score |
| Nicolas *et al*. J Pediatrics | 2022 | Retrospective | 97 | 0 – 18 year olds | Treatment success |
| Petrosyan *et al.* J Ped Surg | 2022 | Retrospective | 43 | 2 - 18 year olds | None |
| Keane *et al*. J Ped Surg | 2022 | Prospective | 22 | 0 – 18 year olds | None |
| Jarzebicka *et al*. J Clinical Med | 2021 | Retrospective | 60 | 1 - 17 years old | None |
| Idrissa *et al*. African J Ped Surg | 2021 | Retrospective | 14 | 0 - 9 years old | None |
| Chams Anturi *et al*. J Lap Adv Surg Tech | 2021 | Retrospective | 21 | 0 - 16 year olds | None |
| Bahadir *et al.* J Behcet Uz Child Hosp | 2021 | Retrospective | 12 | 1 - 17 years old | None |
| Nabi *et al*. J Ped Surg | 2020 | Retrospective | 17 | 4 - 18 year olds | Eckardt score |
| Wood *et al*. J Ped Surg | 2020 | Prospective | 21 | 2 - 17 year olds | None |
| Liu *et al*. J Gastroent | 2020 | Retrospective | 130 | 0 - 17 years old | Eckardt score |
| Chone *et al*. J Ped Gastroent Nutrition | 2019 | Retrospective | 117 | 0 – 18 year olds | Eckardt score |
| Nabi *et al*. Surgical Endoscopy | 2019 | Retrospective | 44 | 4 - 18 year olds | None |
| Yu *et al*. J Ped Surg | 2019 | Retrospective | 12 | 0 – 17 year olds | None |
| Vandewalle *et al*. J Surg Research | 2018 | Retrospective | 26 | 0 – 17 year olds | None |
| Kethman *et al*. J Ped Surg | 2018 | Prospective | 10 | 7 - 17 year olds | None |
| Miao *et al*. J Ped Gastroent Nutrition | 2018 | Retrospective | 21 | 0 – 18 year olds | None |
| Saliakellis *et al*. Eur J Ped | 2017 | Retrospective | 48 | 3 - 17 year olds | None |
| Grabowski *et al*. Gastroent Rev | 2017 | Retrospective | 11 | 6 - 17 year olds | None |
| Meyer *et al*. J Gastroent Hepat | 2016 | Retrospective | 42 | 0 – 18 year olds | None |
| Nabi *et al*. J Neurogastroent Motility | 2016 | Retrospective | 15 | 9 - 18 year olds | None |
| Tan *et al*. J Ped Surg | 2016 | Retrospective | 21 | 6 - 17 year olds | Eckardt score |
| Zagory *et al*. J Lap Adv Surg Tech | 2016 | Retrospective | 23 | 0 - 17 year olds | None |
| Smits *et al.* J Pediatrics | 2016 | Retrospective | 87 | 0 – 17 year olds | None |
| Erginel *et al*. Acta Surg Belgica | 2016 | Retrospective | 22 | 0 - 17 years old | None |
| Caldaro *et al*. J Ped Surg | 2015 | Retrospective | 18 | 2 - 17 year olds | None |
| Chen *et al*. Gastroint Endoscopy | 2015 | Prospective | 27 | 6 - 17 year olds | Eckardt score |
| Li *et al*. J Ped Surg | 2015 | Prospective | 9 | 10 - 17 year olds | Eckardt score |
| Pachl *et al*. J Ped Surg | 2014 | Retrospective | 28 | 3 - 17 year olds | None |
| Ashraf *et al.* Pakistan J Med Health Sci | 2014 | Retrospective | 10 | 0 - 4 years old | None |
| Esposito *et al*. J Lap Adv Surg Tech | 2013 | Retrospective | 31 | 5 - 14 year olds | None |
| Hallal *et al*. Ped Surg Int | 2012 | Retrospective | 13 | 1 - 14 year olds | None |
| Di Nardo *et al*. Gastroint Endoscopy | 2012 | Prospective | 24 | 5 - 17 year olds | Eckardt score |
| Marlais *et al*. J Ped Child Health | 2011 | Prospective | 17 | 5 - 18 year olds | Quality of life |
| Tannuri *et al*. J Ped Surg | 2010 | Retrospective | 15 | 9 - 17 year olds | None |
| Jung *et al*. Gastroent Clin Bio | 2010 | Retrospective | 22 | 0 - 16 year olds | None |
| Corda *et al*. Surg Endoscopy | 2010 | Retrospective | 20 | 5 - 15 year olds | None |
| Logan *et al*. J Lap Adv Surg Tech | 2009 | Retrospective | 12 | 10 - 17 year olds | Post-op manometry |
| Pastor *et al*. J Ped Surg | 2009 | Retrospective | 40 | 0 – 18 year olds | Treatment failure |
| Askegard-Giesmann *et al*. J Ped Surg | 2009 | Retrospective | 26 | 0 – 18 year olds | None |
| Zhang *et al*. World J Ped | 2009 | Retrospective | 13 | 3 - 14 year olds | None |
| Vaos *et al*. J Ped Surg | 2008 | Retrospective | 15 | 6 - 13 year olds | None |
| Garzi *et al*. J Ped Gastro Nutrition | 2007 | Retrospective | 12 | 3 - 7 year olds | None |
| Mattioli *et al*. Surg Endo Intervent Tech | 2003 | Retrospective | 20 | 5 - 14 year olds | None |
| Hussain *et al.* Digestive Dis Sci | 2002 | Retrospective | 33 | 0 - 16 year olds | None |
| Khan *et al*. Disease Esophagus | 2002 | Prospective | 12 | 3 - 12 year olds | None |
| Karnak *et al*. Eur J Ped Surg | 2001 | Retrospective | 20 | 2 - 15 year olds | None |
| Patti *et al*. J Ped Surg | 2001 | Retrospective | 13 | 6 - 17 year olds | None |
| Esposito *et al.* J Ped Surg | 2000 | Retrospective | 10 | 2 - 13 year olds | None |
| Hamza *et al*. Eur J Ped Surg | 1999 | Retrospective | 11 | 1 - 14 year olds | None |
| Tovar *et al*. J Ped Surg | 1998 | Retrospective | 18 | 0 – 17 year olds | None |
| Morris-Stiff *et al*. Annals RCS | 1997 | Retrospective | 10 | 0 – 18 year olds | None |
| Lelli *et al*. J Ped Surg | 1997 | Retrospective | 19 | 1 - 17 years old | None |
| Avanoglu *et al*. Ped Surg Int | 1996 | Retrospective | 9 | 0 - 14 years old | None |
| Illi *et al*. Eur J Ped Surg | 1994 | Retrospective | 16 | 1 - 15 years old | None |
| Emblem *et al*. Archives Dis Childhood | 1993 | Retrospective | 14 | 0 - 10 years old | None |
