# Supplementary material for: Outcome reporting in studies of paediatric achalasia: A systematic review
Source: J Pediatr Gastroenterol Nutr. 2025 Jun 22;81(3):523–9. doi: 10.1002/jpn3.70128 (PMC12408982; doi:10.1002/jpn3.70128)
Supplement: Supplementary file 3 — Table S3: Variation in reported outcome definitions and measures between studies. [file JPN3-81-523-s003.docx]

Supplementary Table 2: Variation in reported outcome definitions and measures between studies.

| **Reported Outcome** | **Definitions/Measures** |
| --- | --- |
| Symptom improvement or recurrence | Sustained relief of symptoms or dysphagia without the necessity for further medical intervention (Nicolas, Aumar et al. 2022; Delgado-Miguel, Amarnath et al. 2024)  Presence (persistence or recurrence) of any symptom(s) post-intervention (patient and/or parent reported)(Emblem, Stringer et al. 1993, Avanoğlu and Mutaf 1996, Morris-Stiff, Khan R Fau - Foster et al. 1997, Tovar, Prieto et al. 1998, Hamza, Awad et al. 1999, Esposito, Mendoza-Sagaon et al. 2000, Karnak, Senocak et al. 2001, Patti, Albanese et al. 2001, Hussain, Thomas et al. 2002, Khan, Shah et al. 2002, Garzi, Valla et al. 2007, Askegard-Giesmann, Grams et al. 2009, Logan, Vossoughi et al. 2009, Corda, Pacilli et al. 2010, Tannuri, Tannuri et al. 2010, Hallal, Kieling et al. 2012, Ashraf, Kayani et al. 2014, Pachl, Rex et al. 2014, Erginel, Gun Soysal et al. 2016, Smits, van Lennep et al. 2016, Zagory, Golden et al. 2016, Grabowski, Korlacki et al. 2017, Vandewalle, Frye et al. 2018, Bahadır, Ergun et al. 2021, Idrissa, Oumarou et al. 2021, Jarzębicka, Czubkowski et al. 2021, Saiad, Idrissi Bahre et al. 2023)  Eckardt score ≤3 and no need for further intervention (Wood, Chandler et al. 2020)  Symptoms occurring daily, consistent with achalasia, interfering with quality of life (Meyer, Catto-Smith et al. 2017)  Free of symptoms (success) or improvement (partial) (Jung, Michaud et al. 2010)  Modified Visick symptom scale (Mattioli, Espocito et al. 2003) |
| Quality of life | PedsQL(Marlais, Fishman et al. 2011, Yu, Rosenfeld et al. 2019, Saiad, Idrissi Bahre et al. 2023)  Kidscreen-52(Smits, van Lennep et al. 2016)  Short Form Health Survey (SF-36)(Smits, van Lennep et al. 2016)  Gastrointestinal PedsQL Symptoms Scales and Module(Saiad, Idrissi Bahre et al. 2023)  Achalasia Severity Questionnaire(Yu, Rosenfeld et al. 2019, Jarzębicka, Czubkowski et al. 2021)  Adult disease-specific quality of life assessment(Smits, van Lennep et al. 2016)  Disease-specific quality of life for children with achalasia(Marlais, Fishman et al. 2011, Smits, van Lennep et al. 2016)  Pediatric GORD Symptom and Quality of Life Questionnaire(Yu, Rosenfeld et al. 2019)  “Impact on quality of life” assessed via questionnaire(Meyer, Catto-Smith et al. 2017) |
| Technical procedure success | Completion of Heller’s cardiomyotomy laparoscopically(Patti, Albanese et al. 2001, Chams Anturi, Romero Espitia et al. 2021, Provenzano, Pulvirenti et al. 2023)  Completion of POEM without need for conversion to surgery(Wood, Chandler et al. 2020, Petrosyan, Mostammand et al. 2022, Samejima, Yoshimura et al. 2023)  Ability to complete POEM procedure(Choné, Familiari et al. 2019, Nabi, Ramchandani et al. 2019, Liu, Wang et al. 2020) |
| Post-operative manometry | Intra-operatively post-myotomy – change in LOS pressure(Yu, Rosenfeld et al. 2019)  Routine at 1 month post-procedure – change in LOS pressure and IRP(Chen, Li et al. 2015)  Routine at 3 months post-procedure – change in LOS pressure and IRP(Li, Tan et al. 2015)  Routine at 6 months post-procedure – change in LOS pressure and IRP(Caldaro, Familiari et al. 2015, Provenzano, Pulvirenti et al. 2023)  Routine at 12 months post-procedure – change in LOS pressure and IRP(Nabi, Ramchandani et al. 2016, Miao, Wu et al. 2018, Nabi, Ramchandani et al. 2019)  Routine at 6 – 12 months and 5 – 15 years post-procedure – change in LOS and oesophageal pressure(Vaos, Demetriou et al. 2008)  Routine post-procedure, time not specified – change in LOS pressure and IRP(Tovar, Prieto et al. 1998, Hamza, Awad et al. 1999, Esposito, Mendoza-Sagaon et al. 2000, Logan, Vossoughi et al. 2009, Nabi, Ramchandani et al. 2020)  Screening in patients with symptoms(Avanoğlu and Mutaf 1996, Patti, Albanese et al. 2001, Garzi, Valla et al. 2007) |
| Post-operative contrast study | Post-operative to screen for a leak(Esposito, Mendoza-Sagaon et al. 2000, Ashraf, Kayani et al. 2014, Nabi, Ramchandani et al. 2019, Nabi, Ramchandani et al. 2020, Provenzano, Pulvirenti et al. 2023)  Screening at 2 months post-procedure (>10 years old) or if symptomatic (<10 years old)(Provenzano, Pulvirenti et al. 2023)  Screening between 1 and 5 months post-procedure(Karnak, Senocak et al. 2001)  Screening in patients with symptoms(Emblem, Stringer et al. 1993, Morris-Stiff, Khan R Fau - Foster et al. 1997, Hamza, Awad et al. 1999, Garzi, Valla et al. 2007, Caldaro, Familiari et al. 2015, Vandewalle, Frye et al. 2018, Saiad, Idrissi Bahre et al. 2023, Delgado-Miguel, Amarnath et al. 2024)  Screening at 6 months post-procedure(Esposito, Mendoza-Sagaon et al. 2000)  Screening at 12 months post-procedure(Nabi, Ramchandani et al. 2019, Chams Anturi, Romero Espitia et al. 2021)  Screening at 1, 3, 6 and 12 months post-procedure(Miao, Wu et al. 2018, Nabi, Ramchandani et al. 2020)  Screening post-procedure, time not specified(Avanoğlu and Mutaf 1996, Lelli, Drongowski et al. 1997)  Measurement of oesophageal diameter post-procedure(Vaos, Demetriou et al. 2008, Di Nardo, Rossi et al. 2012, Tan, Zhu et al. 2016)  Measurement of height and width of barium column post-procedure(Khan, Shah et al. 2002) |
| Post-operative pH study | Screening at 3 months post-procedure(Nabi, Ramchandani et al. 2019)  Screening at 6 months post-procedure – abnormal if acid exposure time >6%(Provenzano, Pulvirenti et al. 2023)  Screening at 6 months post-procedure - Boix-Ochoa score(Vaos, Demetriou et al. 2008)  Screening post-procedure, time not specified(Avanoğlu and Mutaf 1996)  Screening in patients with symptoms suggestive of GORD(Saliakellis, Thapar et al. 2017) |
| Post-operative endoscopy | Screening for complications at 24 – 48 hours post-procedure(Caldaro, Familiari et al. 2015)  Screening for complications at 3 months post-procedure(Nabi, Ramchandani et al. 2019)  Screening for complications 12 months post-procedure(Vaos, Demetriou et al. 2008, Nabi, Ramchandani et al. 2020, Petrosyan, Mostammand et al. 2022, Provenzano, Pulvirenti et al. 2023)  Screening for complications at 3 and 12 months post-procedure(Samejima, Yoshimura et al. 2023)  Screening for complications at 1, 3, 6 and 12 months post-procedure(Li, Tan et al. 2015, Tan, Zhu et al. 2016, Miao, Wu et al. 2018)  Screening for complications if post-procedure Eckardt score >3(Wood, Chandler et al. 2020)  Screening for complications in symptomatic patients(Avanoğlu and Mutaf 1996, Morris-Stiff, Khan R Fau - Foster et al. 1997, Saliakellis, Thapar et al. 2017) |
| Development of GORD | DeMeester score on ambulatory pH monitoring(Nabi, Ramchandani et al. 2019, Nabi, Ramchandani et al. 2020)  Distal oesophageal acid exposure time >6%(Provenzano, Pulvirenti et al. 2023)  Diagnosis on pH study(Avanoğlu and Mutaf 1996, Tovar, Prieto et al. 1998, Patti, Albanese et al. 2001, Mattioli, Esposito et al. 2003, Corda, Pacilli et al. 2010, Caldaro, Familiari et al. 2015, Saliakellis, Thapar et al. 2017, Nicolas, Aumar et al. 2022)  Diagnosis on endoscopy(Chen, Li et al. 2015, Samejima, Yoshimura et al. 2023)  Patient-reported symptoms(Emblem, Stringer et al. 1993, Lelli, Drongowski et al. 1997, Hamza, Awad et al. 1999, Karnak, Senocak et al. 2001, Hussain, Thomas et al. 2002, Logan, Vossoughi et al. 2009, Pastor, Mills et al. 2009, Zhang, Xu et al. 2009, Tannuri, Tannuri et al. 2010, Pachl, Rex et al. 2014, Chen, Li et al. 2015, Nabi, Ramchandani et al. 2016, Grabowski, Korlacki et al. 2017, Yu, Rosenfeld et al. 2019, Wood, Chandler et al. 2020, Chams Anturi, Romero Espitia et al. 2021, Idrissa, Oumarou et al. 2021)  GerdQ assessment(Liu, Wang et al. 2020)  Reflux Disease Questionnaire(Smits, van Lennep et al. 2016) |
| Development of dysphagia | Reported by patient(Avanoğlu and Mutaf 1996, Hamza, Awad et al. 1999, Vaos, Demetriou et al. 2008, Logan, Vossoughi et al. 2009, Pastor, Mills et al. 2009, Tannuri, Tannuri et al. 2010, Chams Anturi, Romero Espitia et al. 2021, Saiad, Idrissi Bahre et al. 2023)  Contrast study(Karnak, Senocak et al. 2001, Mattioli, Esposito et al. 2003, Esposito, Riccipetitoni et al. 2013)  Endoscopy(Karnak, Senocak et al. 2001) |
| Development of oesophagitis | Los Angeles classification(Nabi, Ramchandani et al. 2016, Tan, Zhu et al. 2016, Choné, Familiari et al. 2019, Nabi, Ramchandani et al. 2019, Nabi, Ramchandani et al. 2020, Samejima, Yoshimura et al. 2023)  Savary-Miller criteria(Vaos, Demetriou et al. 2008)  Appearance at endoscopy not otherwise specified(Avanoğlu and Mutaf 1996) |
| Time/delay to diagnosis | Time from start of symptoms to initial treatment(Vaos, Demetriou et al. 2008, Saliakellis, Thapar et al. 2017)  Time from start of symptoms to diagnosis(Illi and Stauffer 1994, Tannuri, Tannuri et al. 2010, Saiad, Idrissi Bahre et al. 2023) |
| Patient satisfaction | Subjective assessment of outcome (patient pleased with outcome)(Vaos, Demetriou et al. 2008)  Subjective ranking of outcome (excellent, good, fair, poor)(Lelli, Drongowski et al. 1997) |

GORD – gastro-oesophageal reflux disease, POEM – per oral endoscopic myotomy, LOS – lower oesophageal sphincter, IRP – integrated relaxation pressure.

Ashraf, M., M. Kayani and M. Khan (2014). "Early Childhood Achalasia Cardia: a local experience." Pakistan Journal of Medical and Health Sciences **8**: 744 - 746.

Askegard-Giesmann, J. R., J. M. Grams, A. M. Hanna, C. W. Iqbal, S. Teh and C. R. Moir (2009). "Minimally invasive Heller's myotomy in children: safe and effective." Journal of Pediatric Surgery **44**(5): 909-911.

Avanoğlu, A. and O. Mutaf (1996). "Surgical treatment of achalasia in children: is an added antireflux procedure necessary?" Pediatric Surgery International **11**(2): 134-136.

Bahadır, K., E. Ergun, A. Jafarov, M. Bülbül, G. Göllü, M. Bingöl Koloğlu, A. Yağmurlu, A. M. Çakmak and U. Ateş (2021). "Long-Term Follow-up Results of Children Undergoing Achalasia Surgery." Journal of Behcet Uz Children's Hospital **11**(3): 309-313.

Caldaro, T., P. Familiari, E. F. Romeo, G. Gigante, M. Marchese, A. C. I. Contini, G. Federici di Abriola, S. Cucchiara, P. De Angelis, F. Torroni, L. Dall’Oglio and G. Costamagna (2015). "Treatment of esophageal achalasia in children: Today and tomorrow." Journal of Pediatric Surgery **50**(5): 726-730.

Chams Anturi, A., W. Romero Espitia, A. Loockhartt, M. D. Moreno Villamizar, M. Pedraza Ciro, J. E. Villamizar, L. F. Cabrera, N. J. Tinoco Guzman, J. Beltrán, F. Fierro, A. Holguin, A. Silvia, C. Giraldo and M. Rodriguez (2021). "Multicenter Evaluation with Eckardt Score of Laparoscopic Management with Heller Myotomy and Dor Fundoplication for Esophageal Achalasia in a Pediatric Population in Colombia." Journal of Laparoendoscopic & Advanced Surgical Techniques **31**(2): 230-235.

Chen, W.-F., Q.-L. Li, P.-H. Zhou, L.-Q. Yao, M.-D. Xu, Y.-Q. Zhang, Y.-S. Zhong, L.-L. Ma, W.-Z. Qin, J.-W. Hu, M.-Y. Cai, M.-J. He and Z. Cui (2015). "Long-term outcomes of peroral endoscopic myotomy for achalasia in pediatric patients: a prospective, single-center study." Gastrointestinal Endoscopy **81**(1): 91-100.

Choné, A., P. Familiari, B. von Rahden, P. Desai, H. Inoue, Y. Shimamura, N. Eleftheriadis, K. Yamashita, M. A. Khashab, H. Shiwaku, S. Seewald, P. V. Draganov, L. B. M. Alvarez, S. Chaussade, M. Tantau, M. Abraham, J. Marks, G. Arevalo, E. Albéniz, F. Mion, S. Roman, J. Rivory, R. Dubois, A. Lachaux, N. Benech, F. Subtil, T. Ponchon, M. Barret and M. Pioche (2019). "Multicenter Evaluation of Clinical Efficacy and Safety of Per-oral Endoscopic Myotomy in Children." Journal of Pediatric Gastroenterology and Nutrition **69**(5): 523-527.

Corda, L., M. Pacilli, S. Clarke, J. M. Fell, D. Rawat and M. Haddad (2010). "Laparoscopic oesophageal cardiomyotomy without fundoplication in children with achalasia: a 10-year experience." Surgical Endoscopy **24**(1): 40-44.

Delgado-Miguel, C., R. P. Amarnath and J. I. Camps (2024). "Robotic-assisted vs. Laparoscopic Heller's Myotomy for Achalasia in Children." Journal of Pediatric Surgery **59**(6): 1072-1076.

Di Nardo, G., P. Rossi, S. Oliva, M. Aloi, D. A. Cozzi, S. Frediani, A. Redler, S. Mallardo, F. Ferrari and S. Cucchiara (2012). "Pneumatic balloon dilation in pediatric achalasia: efficacy and factors predicting outcome at a single tertiary pediatric gastroenterology center." Gastrointestinal Endoscopy **76**(5): 927-932.

Emblem, R., M. D. Stringer, C. M. Hall and L. Spitz (1993). "Current results of surgery for achalasia of the cardia." Archives of Disease in Childhood **68**(6): 749.

Erginel, B., F. Gun Soysal, E. Keskin, A. Celik and T. Salman (2016). "Early myotomy and fundoplication in achalasia in childhood: a single-centre experience for 22 years." Acta Chirurgica Belgica **116**(1): 16-18.

Esposito, C., M. Mendoza-Sagaon, B. Roblot-Maigret, G. Amici, P. Desruelle and P. Montupet (2000). "Complications of laparoscopic treatment of esophageal achalasia in children." Journal of Pediatric Surgery **35**(5): 680-683.

Esposito, C., G. Riccipetitoni, S. F. Chiarenza, A. Roberti, C. Vella, F. Alicchio, G. Fava, M. Escolino, T. De Pascale and A. Settimi (2013). "Long-Term Results of Laparoscopic Treatment of Esophageal Achalasia in Children: A Multicentric Survey." Journal of Laparoendoscopic & Advanced Surgical Techniques **23**(11): 955-959.

Garzi, A., J. S. Valla, F. Molinaro, G. Amato and M. Messina (2007). "Minimally Invasive Surgery for Achalasia: Combined Experience of Two European Centers." Journal of Pediatric Gastroenterology and Nutrition **44**(5): 587-591.

Grabowski, A., W. Korlacki, M. Pasierbek, R. Pułtorak, F. Achtelik and M. Ilewicz (2017). "Pediatric achalasia. Single-center study of interventional treatment." Gastroenterology Review/Przegląd Gastroenterologiczny **12**(2): 98-104.

Hallal, C., C. O. Kieling, D. L. Nunes, C. T. Ferreira, G. Peterson, S. G. S. Barros, C. A. Arruda, J. C. Fraga and H. A. S. Goldani (2012). "Diagnosis, misdiagnosis, and associated diseases of achalasia in children and adolescents: a twelve-year single center experience." Pediatric Surgery International **28**(12): 1211-1217.

Hamza, A. F., H. A. Awad and O. Hussein (1999). "Cardiac Achalasia in Children. Dilatation or Surgery?" Eur J Pediatr Surg **9**(05): 299-302.

Hussain, S. Z., R. Thomas and V. Tolia (2002). "A Review of Achalasia in 33 Children." Digestive Diseases and Sciences **47**(11): 2538-2543.

Idrissa, S., A. Oumarou, A. Mahmoudi, A. Elmadi, K. Khattala and Y. Bouabdallah (2021). "Diagnosis and surgical management of children with oesophageal achalasia: A 10-year single-centre experience in Morocco." African Journal of Pediatric Surgery(0974-5998 (Electronic)).

Illi, O. E. and U. G. Stauffer (1994). "Achalasia in Childhood and Adolescence." Eur J Pediatr Surg **4**(04): 214-217.

Jarzębicka, D., P. Czubkowski, J. Sieczkowska-Gołub, J. Kierkuś, A. Kowalski, M. Stefanowicz and G. Oracz (2021) "Achalasia in Children—Clinical Presentation, Diagnosis, Long-Term Treatment Outcomes, and Quality of Life." Journal of Clinical Medicine **10** DOI: 10.3390/jcm10173917.

Karnak, I., M. E. Senocak, F. C. Tanyel and N. Büyükpamukçu (2001). "Achalasia in childhood: surgical treatment and outcome." European journal of pediatric surgery : official journal of Austrian Association of Pediatric Surgery ... [et al] = Zeitschrift fur Kinderchirurgie **11**(4): 223-229.

Khan, A. A., S. W. H. Shah, A. Alam, A. K. Butt and F. Shafqat (2002). "Efficacy of Rigiflex balloon dilatation in 12 children with achalasia: a 6-month prospective study showing weight gain and symptomatic improvement." Diseases of the esophagus : official journal of the International Society for Diseases of the Esophagus **15**(2): 167-170.

Lelli, J. L., R. A. Drongowski and A. G. Coran (1997). "Efficacy of the transthoracic modified Heller myotomy in children with achalasia—A 21-year experience." Journal of Pediatric Surgery **32**(2): 338-341.

Li, C., Y. Tan, X. Wang and D. Liu (2015). "Peroral endoscopic myotomy for treatment of achalasia in children and adolescents." Journal of Pediatric Surgery **50**(1): 201-205.

Liu, Z., Y. Wang, Y. Fang, Y. Huang, H. Yang, X. Ren, M. Xu, S. Chen, W. Chen, Y. Zhong, Y. Zhang, W. Qin, J. Hu, M. Cai, L. Yao, Q. Li and P. Zhou (2020). "Short-term safety and efficacy of peroral endoscopic myotomy for the treatment of achalasia in children." Journal of Gastroenterology **55**(2): 159-168.

Logan, M. S., F. Vossoughi, C. M. Watson, R. Amarnath and J. I. Camps (2009). "A Novel Technique for the Surgical Treatment of Achalasia in Children: Evaluated with Postoperative Esophageal Manometry." Journal of Laparoendoscopic & Advanced Surgical Techniques **19**(4): 589-593.

Marlais, M., J. R. Fishman, J. M. E. Fell, D. J. Rawat and M. J. Haddad (2011). "Health-related quality of life in children with achalasia." Journal of Paediatrics and Child Health **47**(1-2): 18-21.

Mattioli, G., C. Esposito, A. P. Prato, P. Doldo, M. Castagnetti, A. Barabino, P. Gandullia, A. M. Staiano, A. Settimi, S. Cucchiara, G. Montobbio and V. Jasonni (2003). "Results of the laparoscopic Heller-Dor procedure for pediatric esophageal achalasia." Surgical Endoscopy And Other Interventional Techniques **17**(10): 1650-1652.

Meyer, A., A. Catto-Smith, J. Crameri, D. Simpson, G. Alex, W. Hardikar, D. Cameron and M. Oliver (2017). "Achalasia: Outcome in children." Journal of Gastroenterology and Hepatology **32**(2): 395-400.

Miao, S., J. Wu, J. Lu, Y. Wang, Z. Tang, Y. Zhou, Z. Huang, H. Ying and P. Zhou (2018). "Peroral Endoscopic Myotomy in Children With Achalasia." Journal of Pediatric Gastroenterology and Nutrition **66**(2): 257-262.

Morris-Stiff, G., M. E. Khan R Fau - Foster, J. Foster Me Fau - Lari and J. Lari (1997). "Long-term results of surgery for childhood achalasia." Annals of the Royal College of Surgeons(0035-8843 (Print)).

Nabi, Z., M. Ramchandani, R. Chavan, S. Darisetty, R. Kalapala, U. Shava, M. Tandan, R. Kotla and D. N. Reddy (2019). "Outcome of peroral endoscopic myotomy in children with achalasia." Surgical Endoscopy **33**(11): 3656-3664.

Nabi, Z., M. Ramchandani, S. Darisetty, R. Kotla and D. N. Reddy (2020). "Impact of prior treatment on long-term outcome of peroral endoscopic myotomy in pediatric achalasia." Journal of Pediatric Surgery **55**(8): 1552-1555.

Nabi, Z., M. Ramchandani, D. N. Reddy, S. Darisetty, R. Kotla, R. Kalapala and R. Chavan (2016). "Per Oral Endoscopic Myotomy in Children with Achalasia Cardia." Journal of Neurogastroenterology and Motility **22**(4): 613-619.

Nicolas, A., M. Aumar, L. C. Tran, A. Tiret, R. Duclaux-Loras, L. Bridoux-Henno, F. Campeotto, A. Fabre, A. Breton, J. Languepin, M. Kyheng, J. Viala, S. Coopman and F. Gottrand (2022). "Comparison of Endoscopic Dilatation and Heller's Myotomy for Treating Esophageal Achalasia in Children: A Multicenter Study." The Journal of Pediatrics **251**: 134-139.e132.

Pachl, M. J., D. Rex, P. DeCoppi, K. Cross, E. M. Kiely, D. Drake, A. Pierro and J. I. Curry (2014). "Paediatric laparoscopic Heller’s cardiomyotomy: A single centre series." Journal of Pediatric Surgery **49**(2): 289-292.

Pastor, A. C., J. Mills, M. A. Marcon, S. Himidan and P. C. W. Kim (2009). "A single center 26-year experience with treatment of esophageal achalasia: is there an optimal method?" Journal of Pediatric Surgery **44**(7): 1349-1354.

Patti, M. G., C. T. Albanese, G. W. Holcomb, D. Molena, P. M. Fisichella, S. Perretta and L. W. Way (2001). "Laparoscopic heller myotomy and dor fundoplication for esophageal achalasia in children." Journal of Pediatric Surgery **36**(8): 1248-1251.

Petrosyan, M., S. Mostammand, A. A. Shah, A. Darbari and T. D. Kane (2022). "Per Oral Endoscopic Myotomy (POEM) for pediatric achalasia: Institutional experience and outcomes." Journal of Pediatric Surgery **57**(11): 728-735.

Provenzano, L., R. Pulvirenti, M. Duci, G. Capovilla, A. Costantini, F. Forattini, P. Gamba, M. Costantini, F. Fascetti-Leon and e. Salvador (2023). "Laparoscopic Heller–Dor Is a Persistently Effective Treatment for Achalasia Even in Pediatric Patients: A 25-Year Experience at a Single Tertiary Center." Eur J Pediatr Surg **33**(06): 493-498.

Saiad, M. O., M. N. Idrissi Bahre and N. Ryad (2023). "Quality-of-life Assessment among Children with Esophageal Achalasia." Journal of Indian Association of Pediatric Surgeons **28**(6).

Saliakellis, E., N. Thapar, D. Roebuck, F. Cristofori, K. Cross, E. Kiely, J. Curry, K. Lindley and O. Borrelli (2017). "Long-term outcomes of Heller’s myotomy and balloon dilatation in childhood achalasia." European Journal of Pediatrics **176**(7): 899-907.

Samejima, Y., S. Yoshimura, Y. Okata, H. Sakaguchi, H. Abe, S. Tanaka, Y. Kodama and Y. Bitoh (2023). "Peroral Endoscopic Myotomy in Pediatric Patients with Achalasia up to 12 Years of Age: A Pilot Study in a Single-Center Experience in Japan." Eur J Pediatr Surg **34**(01): 097-101.

Smits, M., M. van Lennep, R. Vrijlandt, M. Benninga, J. Oors, R. Houwen, F. Kokke, D. van der Zee, J. Escher, A. van den Neucker, T. de Meij, F. Bodewes, J. Schweizer, G. Damen, O. Busch and M. van Wijk (2016). "Pediatric Achalasia in the Netherlands: Incidence, Clinical Course, and Quality of Life." The Journal of Pediatrics **169**: 110-115.e113.

Tan, Y., H. Zhu, C. Li, Y. Chu, J. Huo and D. Liu (2016). "Comparison of peroral endoscopic myotomy and endoscopic balloon dilation for primary treatment of pediatric achalasia." Journal of Pediatric Surgery **51**(10): 1613-1618.

Tannuri, A. C. A., U. Tannuri, M. C. P. Velhote and R. L. P. Romão (2010). "Laparoscopic extended cardiomyotomy in children: an effective procedure for the treatment of esophageal achalasia." Journal of Pediatric Surgery **45**(7): 1463-1466.

Tovar, J. A., G. Prieto, M. Molina and J. Arana (1998). "Esophageal function in achalasia: Preoperative and postoperative manometric studies." Journal of Pediatric Surgery **33**(6): 834-838.

Vandewalle, R. J., C. C. Frye, M. P. Landman, J. M. Croffie and F. J. Rescorla (2018). "Clinical factors and high-resolution manometry predicting response to surgery for achalasia in children." Journal of Surgical Research **229**: 345-350.

Vaos, G., L. Demetriou, C. Velaoras and C. Skondras (2008). "Evaluating long-term results of modified Heller limited esophagomyotomy in children with esophageal achalasia." Journal of Pediatric Surgery **43**(7): 1262-1269.

Wood, L. S. Y., J. M. Chandler, K. E. Portelli, J. S. Taylor, W. C. Kethman and J. K. Wall (2020). "Treating children with achalasia using per-oral endoscopic myotomy (POEM): Twenty-one cases in review." Journal of Pediatric Surgery **55**(6): 1006-1012.

Yu, Y. R., E. H. Rosenfeld, E. H. Chiou, B. P. Chumpitazi, S. C. Fallon and M. L. Brandt (2019). "High-resolution manometric guidance during laparoscopic Heller myotomy: Impact on quality of life and symptom severity for children with achalasia." Journal of Pediatric Surgery **54**(5): 1063-1068.

Zagory, J. A., J. M. Golden, N. E. Demeter, Y. Nguyen, H. R. Ford and N. X. Nguyen (2016). "Heller Myotomy Is Superior to Balloon Dilatation or Botulinum Injection in Children with Achalasia: A Two-Center Review." Journal of Laparoendoscopic & Advanced Surgical Techniques **26**(6): 483-487.

Zhang, Y., C.-D. Xu, A. Zaouche and W. Cai (2009). "Diagnosis and management of esophageal achalasia in children: analysis of 13 cases." World Journal of Pediatrics **5**(1): 56-59.
