# Supplementary material for: Outcome reporting in studies of paediatric achalasia: A systematic review
Source: J Pediatr Gastroenterol Nutr. 2025 Jun 22;81(3):523–9. doi: 10.1002/jpn3.70128 (PMC12408982; doi:10.1002/jpn3.70128)
Supplement: Supplementary file 4 — Table S4: Results of the COSMIN risk of bias assessment. [file JPN3-81-523-s004.docx]

Supplementary Table 3: Results of the COSMIN risk of bias assessment.

|  | **Samejima 2023** | **Nicolas 2022** | **Nabi 2020** | **Liu 2020** | **Chone 2019** | **Tan 2016** | **Chen 2015** | **Li 2015** | **Di Nardo 2012** | **Marlais 2011** | **Logan 2009** | **Pastor 2009** |
| --- | --- | --- | --- | --- | --- | --- | --- | --- | --- | --- | --- | --- |
| **Mean Score** | 2.2 | 2.7 | 2.4 | 2.1 | 2.4 | 2.6 | 2.3 | 2.3 | 2.0 | 2.0 | 2.4 | 2.7 |
| **PROM design** |  |  |  |  |  |  |  |  |  |  |  |  |
| **1** | 1 | 1 | 1 | 1 | 1 | 1 | 1 | 1 | 1 | 1 | 1 | 1 |
| **2** | 1 | 3 | 1 | 1 | 1 | 1 | 1 | 1 | 1 | 1 | 3 | 3 |
| **3** | 4 | 4 | 4 | 4 | 1 | 4 | 4 | 4 | 1 | 1 | 1 | 1 |
| **4** | 3 | 3 | 1 | 1 | 1 | 3 | 3 | 3 | 1 | 1 | 3 | 4 |
| **5** | 4 | 4 | 4 | 4 | 4 | 4 | 4 | 4 | 4 | 1 | 4 | 4 |
| **13** | 4 | 2 | 2 | 1 | 1 | 4 | 4 | 4 | 4 | 1 | 4 | 3 |
| **14** | 4 | 4 | 4 | 4 | 4 | 4 | 4 | 4 | 4 | 4 | 4 | 4 |
| **Reliability** |  |  |  |  |  |  |  |  |  |  |  |  |
| **1** | 1 | 1 | 2 | 1 | 2 | 2 | 2 | 1 | 1 | 2 | 2 | 3 |
| **2** | 1 | 3 | 1 | 1 | 1 | 3 | 1 | 1 | 1 | 3 | 1 | 3 |
| **3** | 2 | 3 | 2 | 3 | 2 | 2 | 2 | 2 | 1 | 2 | 2 | 3 |
| **5** | 4 | 4 | 4 | 4 | 4 | 4 | 4 | 4 | 4 | 4 | 4 | 4 |
| **8** | 3 | 3 | 3 | 3 | 3 | 3 | 3 | 3 | 3 | 3 | 3 | 3 |
| **Measurement error** |  |  |  |  |  |  |  |  |  |  |  |  |
| **1** | 1 | 1 | 2 | 1 | 2 | 2 | 2 | 1 | 1 | 2 | 2 | 3 |
| **2** | 1 | 3 | 1 | 1 | 1 | 3 | 1 | 1 | 1 | 3 | 1 | 3 |
| **3** | 2 | 3 | 2 | 3 | 3 | 2 | 2 | 2 | 1 | 2 | 2 | 3 |
| **5** | 4 | 4 | 4 | 4 | 4 | 4 | 4 | 4 | 4 | 4 | 4 | 4 |
| **6** | 3 | 3 | 3 | 3 | 3 | 3 | 3 | 3 | 1 | 3 | 3 | 3 |
| **Criterion validity** |  |  |  |  |  |  |  |  |  |  |  |  |
| **1** | NS | NS | NS | NS | NS | NS | NS | NS | NS | NS | NS | NS |
| **2** | NS | NS | NS | NS | NS | NS | NS | NS | NS | NS | NS | NS |
| **3** | 3 | 3 | 3 | 3 | 3 | 3 | 3 | 3 | 3 | 3 | 3 | 3 |
| **Hypotheses testing for construct validity** | | |  |  |  |  |  |  |  |  |  |  |
| **1** | 1 | 1 | 4 | 1 | 4 | 1 | 1 | 1 | 1 | 1 | 1 | 1 |
| **2** | 1 | 3 | 3 | 2 | 3 | 2 | 1 | 2 | 1 | 1 | 1 | 1 |
| **3** | 2 | 2 | 2 | 2 | 2 | 2 | 2 | 2 | 2 | 1 | 2 | 2 |
| **4** | 1 | 3 | 3 | 1 | 3 | 3 | 1 | 1 | 3 | 1 | 3 | 3 |
| **5** | 1 | 2 | 1 | 1 | 2 | 2 | 2 | NS | 2 | 1 | NS | 2 |
| **6** | 2 | 2 | 2 | 2 | 2 | 2 | 2 | NS | 2 | 2 | NS | 2 |
| **7** | 3 | 3 | 3 | 1 | 3 | 3 | 3 | NS | 3 | 3 | NS | 3 |
| **Responsiveness** |  |  |  |  |  |  |  |  |  |  |  |  |
| **8** | 1 | 2 | 1 | 1 | NS | NS | NS | NS | NS | 1 | NS | 2 |
| **9** | 1 | 2 | 2 | 1 | NS | NS | NS | NS | NS | 2 | NS | 2 |
| **10** | 3 | 3 | 1 | 3 | NS | NS | NS | NS | NS | 3 | NS | 3 |
| **11** | NS | NS | NS | NS | 1 | 2 | 1 | 1 | 1 | NS | 1 | NS |
| **12** | NS | NS | NS | NS | 2 | 2 | 1 | 1 | 2 | NS | 2 | NS |
| **13** | NS | NS | NS | NS | 3 | 3 | 3 | 3 | 1 | NS | 3 | NS |

NS – not scored.
